# Feasibility and Safety of Adopting a New Approach in Delivering a 450 nm Blue Laser with a Flattop Beam Profile in Vital Tooth Whitening. A Clinical Case Series with an 8-Month Follow-Up

**DOI:** 10.3390/jcm13020491

**Published:** 2024-01-16

**Authors:** Reem Hanna, Ioana Cristina Miron, Stefano Benedicenti

**Affiliations:** 1 Department of Surgical Sciences and Integrated Diagnostics, University of Genoa, 16132 Genoa, Italy; 5632651@studenti.unige.it (I.C.M.);; 2 Department of Restorative Dental Sciences, UCL-Eastman Dental Institute, Medical School, University College London, London WC1E 6DE, UK; 3 Department of Oral Surgery, King’s College Hospital NHS Foundation Trust, Denmark Hill, London SE5 9RS, UK

**Keywords:** acceptability, BlancOne ULTRA^+^, dental bleaching, dental hypersensitivity, flattop beam, in-office whitening, pain, photoactivation, safety, singlet O_2_

## Abstract

A prospective observational case series included six patients who presented with discoloured upper and lower teeth extending from the right second premolar to the left second premolar. The photoactivation dosimetry and treatment protocol were as follows: λ 450 nm, 1 W, CW; flattop beam profile; 1 cm^2^; 15 J/spot; 10 irradiated spots; an irradiation time of 15 s/spot; three whitening cycles in a single session. Blanc One ULTRA^+^ was the bleaching agent. A visual analogue scale (VAS) was utilised to evaluate the pain intensity and dental hypersensitivity during treatment immediately after complete treatment (T1), 24 h (T2), and 8 h (T3) postoperatively, and at an 8-month follow-up timepoint (T4), whereas the dental colour shade change was assessed using the VITA colour shade guide pre-treatment (T0), T1, and T4. The Gingival index and modified Wong Baker faces scale were utilised to evaluate gingival inflammation and patients’ treatment satisfaction, respectively. Our findings revealed a reduction in the dental colour shade of the six cases between 2 and 10- fold (average of 3.5-fold) at T1 and maintained at T4, indicating significant improvement in the colour shade change with optimal outcomes. The percentage of this improvement for all the patients was ranged between 16.6% and 33.3%. At all timepoints, a “0” score was provided for pain intensity, dental hypersensitivity, and gingival inflammation. Our study demonstrates the feasibility and safety of a λ 450 nm laser delivered with a flattop handpiece to achieve optimal whitening outcomes without adverse effects. This offers a useful guide for dental clinicians for vital in-office tooth whitening. Extensive clinical studies with large data are warranted to validate our study protocol.

## 1. Introduction

In the 21st century, an aesthetically pleasing smile is patients’ highest demand when seeking non-invasive treatments, such as tooth whitening, as an alternative to potentially invasive treatment modalities, such as veneers or crowns, entirely for cosmetic gain [1]. Several studies in the literature have highlighted patients’ desire for pearly white teeth, whereby tooth colour is considered one of the most important key factors for patient satisfaction [2]. White teeth have an impact on social confidence, psychological stability, and, ultimately, quality of life (QoL) [3].

### 1.1. The Photoactivation of Bleaching Agents: Mechanism of Action

Upon absorption of laser energy, hydrogen peroxide (H_2_O_2_) breaks down into water molecules and oxygen radicals, which chemically break down the larger organic pigment molecules (chromophores) in the enamel matrix into smaller, lighter-coloured substances through rapid oxidation [4]. The free radicals open the highlight-pigmented carbon rings and convert them into chains, which are lighter in colour [4].

The generation of free radicals and/or active oxygen via the light or laser irradiation of H_2_O_2_ or sodium hypochlorite has been used for tooth whitening and was investigated using electron spin resonance spectroscopy combined with a spin-trapping technique [5]. When H_2_O_2_ was exposed to light or laser radiation, the amount of hydroxyl radicals generated changed according to the concentration of H_2_O_2_ and irradiation time [5].

The stability of hydrogen peroxide solutions is primarily influenced by temperature, pH value, and, above all, the presence of impurities with a decomposing effect. An increase in temperature promotes decomposition as well as a higher pH value [6,7]. The decomposition of hydrogen peroxide can be initiated with or without the presence of a catalyst. The radicals are formed slowly without the presence of a catalyst in a reaction called self-oxidation-reduction. In the presence of metal ions or enzymes, this reaction can be accelerated. The same is observed with increasing temperatures [6,8]

Oxygen radicals that are released by H_2_O_2_ react with organic chromogens more electively through an oxidising process, breaking strong double bonds, destabilising the chromogenic compound, and ultimately reducing tooth discolouration [9]. The oxidising agent diffuses through the organic matrix of the enamel and dentine, producing the free radicals responsible for the bleaching process (oxidation). When H_2_O_2_ diffuses through the inter-prismatic spaces of the enamel into the tooth, H_2_O_2_ interacts with organic chromophores, resulting in a tooth colour change. This reaction process is influenced by temperature, pH, and light source. H_2_O_2_ is preferred, as it breaks down faster, delivering faster results [10]. The laser light-bleaching gel interaction is associated with photochemical, photo-catalytical, and photothermal activities [11,12].

### 1.2. The Controversial Link between the Bleaching Agent and Its Adverse Effects 

The most commonly available commercial in-office bleaching agent contains H_2_O_2_ at different percentages, ranging from 20% to 40%. It is effective but associated with adverse effects, such as the destruction of the natural organic matrix of enamel and dentine [3,13,14] and an increase in tooth sensitivity, which affects 43–80% of patients after the bleaching procedure with peroxides [13,14,15,16,17]. This effect is most likely due to microscopic surface damage to the enamel, allowing oxygen radicals to diffuse towards the dental nerve and damage cells [18,19,20], which leads to temporary dental nerve inflammation [21]. Nevertheless, the findings in the literature in relation to the effects of bleaching products and concentration on dental hard tissues remain controversial. Several studies revealed no effects on the mechanical properties of dental hard tissues after tooth bleaching [22,23,24,25,26,27,28,29]. Controversially, a number of studies showed the following changes at different concentrations of H_2_O_2_: a decrease in enamel microhardness values [30,31,32,33] and an increase in surface roughness post-bleaching [34,35].

Taking into account the above-mentioned notes, interestingly, it was always thought that H_2_O_2_ concentration is responsible for oral soft and dental hard tissues adverse effects, but several studies have shown that other various parameters play pivotal roles in this regard [36,37,38]. On this note, a recent review conducted by Alkahtani et al., 2020 [39] highlighted the potential factors that can influence the effectiveness of vital dental whitening, such as (1) the concentration/pH of the whitening agent; (2) the method of application and thickness of the bleaching agent on the enamel’s surface; (3) the application duration of the whitening agent; (4) the chemical additives and demineralising agents used; (5) the length of photoactivation; (6) the selective wavelength of irradiation; and (7) the delivery of photonic energy.

### 1.3. Chosen Bleaching Agent Properties 

Taking into account the above-mentioned notes, developing new whitening products and technologies that are efficacious in maximising whitening benefits by accelerating the bleaching reaction and/or minimising reversible/irreversible enamel structural damage and eliminating dental hypersensitivity is important. BlancOne ULTRA^+^ (IDS SpA, Via Valletta San Cristoforo, 28/10, 17100 Savona, Italy) is a whitening agent that contains 35% H_2_O_2_ and photo booster, allowing rapid photochemical activation once irradiated using a suitable light source [40]. An ex vivo study conducted by Pasquale et al., 2023 [40] highlighted that the predictor variable was the patented innovative formulation of photosensitive colourants combined with photons and special energy catalysts in the BlancOne ULTRA^+^ treatment using a solution containing 35% H_2_O_2_, which accelerates oxygen excitation and a release of reactive oxygen species (ROS). Those properties in the bleaching agent offer a strong bleaching potential in a short period of time, which significantly decrease the bleaching application time on the treated teeth, as time plays a vital role in optimising the clinical outcome. Furthermore, the latter study [40] revealed that the pH value of BlancOne ULTRA^+^ was “5.5”, measured with the Five Easy Plus pH Meter FP20-Std-Kit (Mettler Toledo, Columbus, OH, USA). This indicates a high pH, but products with a neutral pH or above the neutrality threshold are balanced with buffer substances capable of raising the pH and keeping it stable over the working time [39]. Hence, it has no impact on changing the tooth’s structure.

Taking into consideration all the above-mentioned properties of BlanceOne ULTRA^+^, this agent was the chosen whitening agent for our study. 

### 1.4. Laser versus Light-Emitted Diode (LED)-Assisted Bleaching

The BlancOne^®^ ARCUS whitening lamp (IDS SpA, Via Valletta San Cristoforo, 28/10, 17100 Savona, Italy) emits blue light (λ 430–490 nm) and is equipped with 10 powerful 5 W LEDs, utilised to activate BlancOne ULTRA^+^ gel, and should be positioned perpendicularly to the teeth at a distance of 5–10 cm from the mouth to ensure good ventilation. Various kinds of luminescent lamps, LEDs, and lasers have been described in the scientific literature, with lasers gaining more attention in recent years [12,41]. As lasers are a much more intense source of energy [42], their role in teeth whitening can accelerate the activation of H_2_O_2_ in the whitening gel and increase the penetration of the dentin surface, thus enhancing the whitening effect by accelerating the release of free radicals within the bleaching gel to decrease the time of the whitening procedure. Laser light is required to activate the chemicals in the whitening gel, producing faster and more effective results.

In summary, laser whitening light has the following three advantages [42]: (1) it shortens the whitening time to 15 s per spot; (2) it shortens the whitening gel resting time to 8 min and 30–45 min for LED cold light bleaching; (3) it causes much less tooth sensitivity to patients, while LED cold light whitening has a strong acid dissolution reaction; and (4) laser teeth whitening works better and lasts longer. Hence, in our study, for the first time, a λ 450 nm blue laser delivered with a flattop beam profile was utilised. Additionally, the flattop beam profile has unique specifications by which the photonic energy is equally distributed over 1 cm^2^, even at distances up to 105 cm from the target [43]. The λ 450 nm blue laser is compatible with a high absorption affinity to the utilised whitening gel, BlancOne ULTRA^+^.

Considering all the scientific controversy in achieving optimal whitening outcomes without adverse effects, studies are required to address the shortfalls and ensure the safety of in-office dental bleaching. Hence, our case series study aimed to evaluate the efficacy and feasibility of a λ 450 nm blue laser delivered with a flattop handpiece to achieve optimal outcomes in vital tooth bleaching in terms of colour shade improvement and its sustainability at an 8-month follow-up timepoint. The primary endpoint was to assess the post-bleaching adverse effects and safety, whereas the secondary endpoint was to evaluate patients’ satisfaction with their whitening treatment. 

Our null hypothesis (H_0_) was that the photoactivation of BlancOne ULTR^+^ with a λ 450 nm blue laser delivered with a flattop handpiece offered no efficiency in terms of colour shade change, colour stability at the 8-month follow-up timepoint, and safety in terms of tooth sensitivity.

## 2. Materials and Methods

### 2.1. Study Design

A prospective observational case series of six patients who presented with discoloured (A2-D2) upper teeth from the upper right second premolar to the upper left second premolar (UR5-UL5) and lower teeth from the lower right second premolar to the lower left second premolar (LR5-LL5). Only living teeth were subjected to the vital whitening process to evaluate the feasibility and safety of the λ 450 nm blue laser (Wiser 3, Doctor Smile-Lambda, Vincenza, Italy) delivered with a flattop handpiece to achieve the photoactivation of BlancOne ULTRA^+^ (the bleaching agent) with optimal outcomes and no adverse effects.

One experienced operator performed all of the cases. As our study was a case series observational study and not a randomised controlled trial or a comparative study, we allocated two independent, experienced assessors (experienced aesthetic dental nurses who were uninvolved in the study) for tooth colour shade evaluation, data collection, and analysis to minimise the interobserver variability and bias. All the data were stored on an Excel spreadsheet.

The study was conducted in accordance with the Declaration of Helsinki. Informed written consent was obtained from all the patients, and a full explanation of the treatment was provided, including a patient information leaflet. Additionally, informed written consent was obtained from all the subjects regarding publishing their clinical photos and our study in a scientific peer-reviewed journal.

### 2.2. Eligibility Criteria

Inclusion Criteria

1. Fit and healthy subjects of both genders aged ≥18 years old presenting with discoloured upper and lower teeth in the region of UR5-UL5 and LR5-LL5, respectively, based on the VITA colour guide (Vita Easyshade^®^; VITA Zahnfabrik H. Rauter GmbH & Co., KG, Bad Säckingen, Germany);
2. Subjects with no active intraoral lesions, active carious lesions, or any previous tooth sensitivity; subjects with external vital tooth discolouration were in the inclusion criteria.

Exclusion Criteria

1. Subjects with allergic reactions to any components in the commercial bleaching agents, such as peroxides, latex, and dyes;
2. Subjects with systematic diseases;
3. Subjects who could not commit to follow-up appointments;
4. Pregnant and lactating women;
5. Subjects with severe periodontal conditions;
6. Subjects who had hypersensitivity to light.

### 2.3. Patient Protocol Prior to Bleaching Treatment

**Figure 1:**
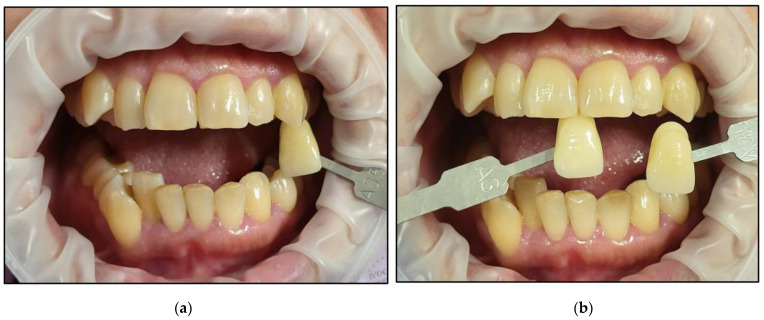
Clinical photos illustrating the process of identifying the darkest colour of the teeth by using the upper left canine as the colour reference. (**a**): the VITA colour shade guide placed adjacent to the upper left canine, as a colour reference to determine the pre-treatment colour shade (A3.5). (**b**): the VITA colour shade placed adjacent to the upper central incisors and the colour shade was A3 compared to upper left canine which was A3.5. Hence, A3.5 was the pre-treatment colour shade.

1. At least one week prior to the treatment, patients underwent a professional dental hygiene session (ultrasonic and guided biofilm therapy protocols);
2. Full medical and social histories were taken from the patient on the day of the bleaching treatment;
3. The oral soft and hard tissues were fully examined to verify any possible problems or risks in performing the bleaching treatment;
4. The patient’s initial colour shade was recorded using the VITA colour guide (Vita Easyshade^®^; VITA Zahnfabrik H. Rauter GmbH & Co., KG, Bad Säckingen, Germany);
5. The colour of a darkened tooth was identified (Figure 1). The upper left canine was used as a colour reference because it is the most saturated tooth in the arch (greater dentin mass and volume of intrinsic pigment). Hence, the canine could be considered a good reference point in selecting a shade because they have the highest chroma (intensity) of the dominant hue (colour) of the teeth [44];
6. Pre-treatment photos were taken;
7. The patients were provided with a full explanation of the bleaching treatment, including all its associated steps. Informed written consent was obtained from all the patients prior to the treatment in relation to the bleaching treatment, photographs, and publishing of the study in a scientific journal.

### 2.4. The Bleaching Agent

#### 2.4.1. BlancOne^®^ ULTRA^+^

The utilised whitening gel in the present study was BlancOne ULTRA^+^ (IDS SpA, Via Valletta San Cristoforo, 28/10, 17100 Savona, Italy), which is ideal for the in-office treatment of the most intrinsic tooth discolouration, such as pharmacological therapies. Blanc One’s ULTRA^+^ formulation contains 35% H_2_O_2_ and three natural photoactivators in synergy that can be photoactivated with a range of wavelengths between λ 430 and λ 490 nm, triggering the efficient release of free radicals and singlet oxygens to allow faster and more effective bleaching results [40]. The whitening agent can be photoactivated with a Blanc One^®^ ARCUS whitening lamp equipped with 10 powerful 5 W LED lights. However, our study, for the first time, utilised a λ 450 nm blue laser energy delivered with a flattop handpiece (Italy) to maximise the whitening results and minimise the bleaching adverse effects (Refer to Section 1.4). Additionally, the utilised [43]. Table 1 shows a brief specification of BlanceOne ULTRA^+^.

#### 2.4.2. BlancOne ULTRA^+^ Preparation

**Figure 2:**
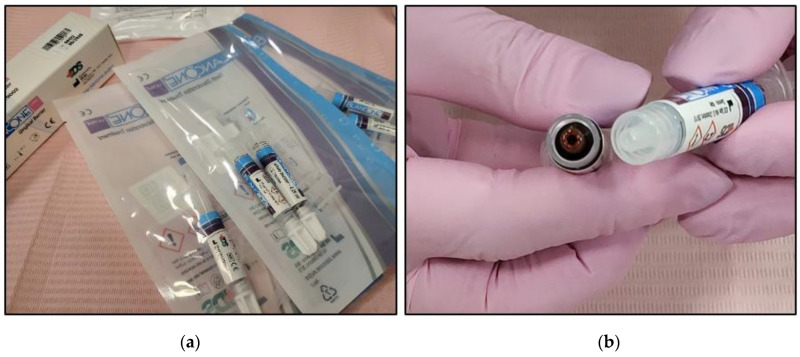
The cycle pack containing the BlancOne ULTRA^+^ gel and its contents. (**a**) The gel cycle pack containing the whitening gel syringe, photo-accelerator gel syringe, connector, and tip for application (**b**).

Each BlancOne ULTRA^+^ cycle pack contains a whitening gel syringe, a photoaccelerator (photo booster) syringe, a connector, and a tip for application (Figure 2a,b).

**Figure 3:**
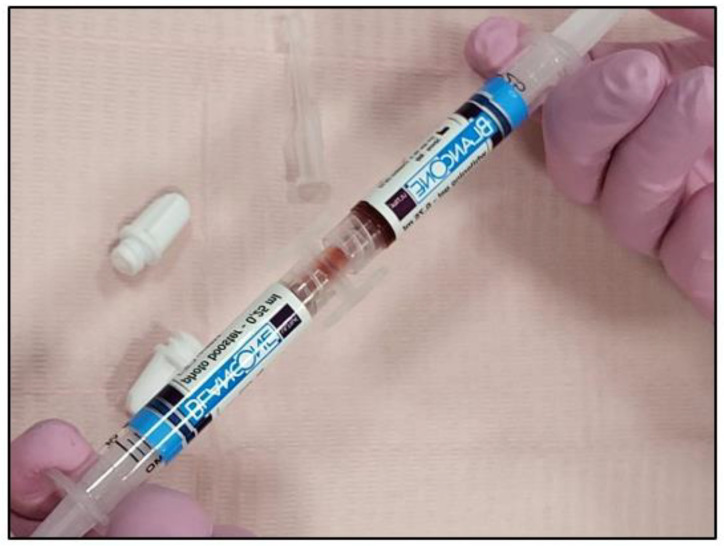
Shows the mixing process of the two BlancOne ULTRA^+^ gel components that are in the two syringes by passing them from one syringe to another back and forth for approximately 20 passages, in order to achieve a homogenous mix.

The steps for BlancOne ULTRA^+^ are outlined below:

1. The two syringes’ caps were removed and then they were connected via a connector;
2. The two components were mixed by transferring them several times from one syringe to the other until a homogeneous mix was achieved (approximately 20 passages back and forth) (Figure 3);
3. The full amount of the mixed gel was transferred into one of the two syringes;
4. Transparent applicator tip was inserted.

The gel mixture was homogenous to ensure maximum bleaching effectiveness. The quantity of gel obtained with each cycle pack was necessary and sufficient for an application. Precaution was necessary to ensure that the gel was not intended for two applications, as this would result in a layer that was too thin. Furthermore, the residual gel begins autoactivation and loses effectiveness between one application and the next. Hence, in our case study, the BlancOne ULTRA^+^ treatment was administered in three whitening cycles during one session.

### 2.5. Steps for the Bleaching Treatment Protocol

#### 2.5.1. Patient Preparation Protocol

**Figure 4:**
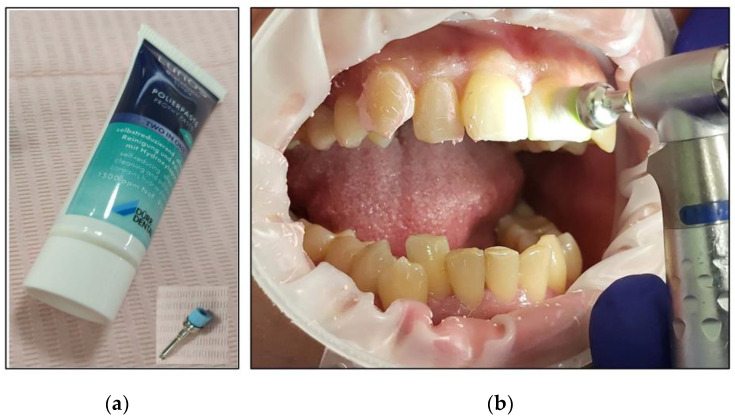
(**a**) The prophylactic paste, Lunos^®^ Prophy Paste Super Soft, and a special brush utilised to remove the salivary biofilm; (**b**) the removal of the salivary biofilm from the required teeth and the direction of the brush perpendicular to the tooth with a circular motion to ensure all the teeth were free from the biofilm.

**Figure 5:**
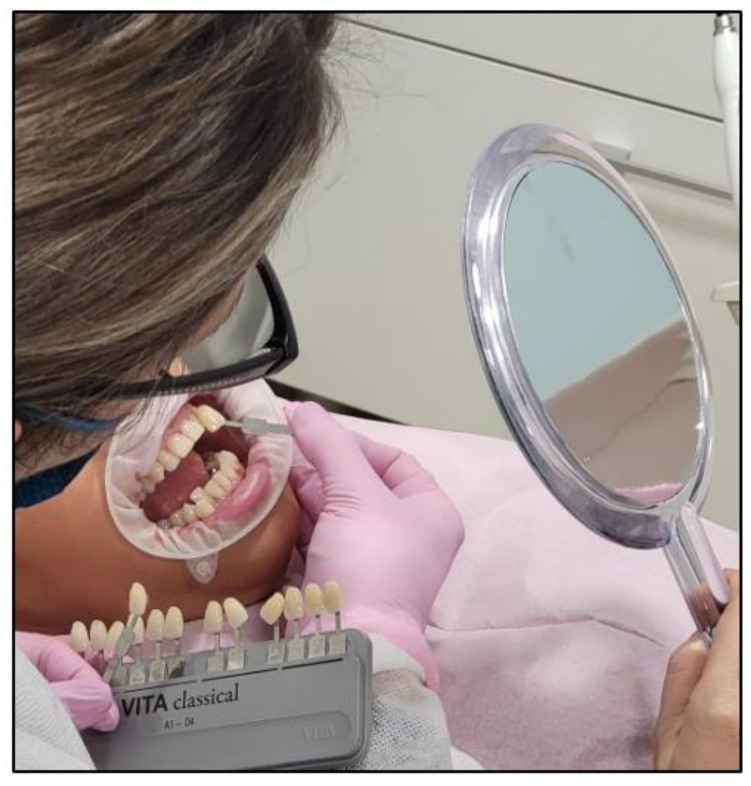
A clinical photo illustrating the process of determining the initial tooth shade.

**Figure 6:**
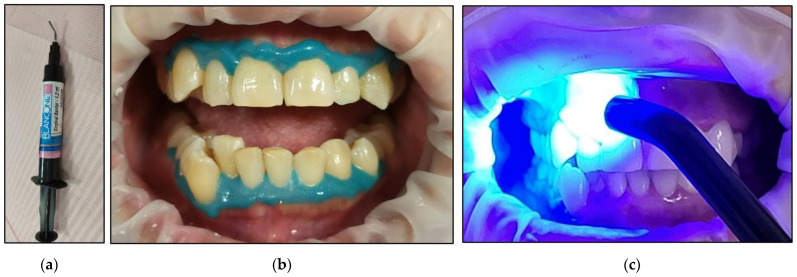
Clinical photos that illustrate the steps of isolating the gingivae of the treated teeth prior bleaching agent application. (**a**): the applicator of the BLANCONE gingival-barrier liquid; (**b**) the gingival-barrier liquid was applied on the tissue of the free gingival margin and the papillae of the treated teeth; (**c**) Photopolymerisation of the gingival-barrier liquid with a Woodpecker LED-B photopolymer lamp.

1. The cheeks and lips were prepared by isolating them with a silicon mouth retractor “OptraGate”-latex-free (Ivovlar Vivadent, Opfikon, Switzerland). OptraGate is a single-use, latex-free lip and cheek retractor that provides increased visibility and accessibility during dental procedures. It is highly flexible and elastic in all directions of movement for patient comfort and assists in keeping the patient’s mouth open;
2. The salivary biofilm was removed from the buccal surfaces of the recruited teeth with a brush and prophylactic paste (Lunos^®^ Prophy Paste Super Soft, DÜRR DENTAL, Kettering, UK) (Figure 4a). The utilised brush (Figure 4a) was applied perpendicular to the tooth surface and rotated to clean in a circulating motion (Figure 4b);
3. The pre-treatment colour shade was identified using the VITA classical A1-D4^®^ shade guide (VITA Zahnfabrik, Bad Säckingen, Germany), which is used especially for the whitening treatment. The tooth shade was placed near the buccal surface of the intended tooth that required bleaching (Figure 5);
4. Pre-treatment photos were taken;
5. Prior to applying the bleaching agent, the gingivae surround UR5-UL5 and LR5-LL5 (i.e., the tissue-free gingival margin and the papillae between the treated teethy) were isolated with a light-curing BLANCONE gingival-barrier liquid dam (BlancOne^®^ CARE, IDS, Savona, Italy) (Figure 6a,b), after achieving a completely dry field. Then, a Woodpecker LED-B photopolymer lamp (Woodpecker, Beijing, China) was used to photopolymerise the gingival barrier (Figure 6c), which took between 20 s and 23 s. Suitable protective eyewear was worn.

#### 2.5.2. Whitening Gel Application and Photoactivation Protocols

**Figure 7:**
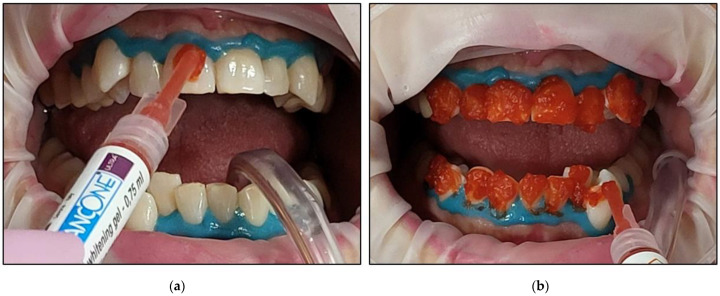
Clinical photos that illustrate the steps for the whitening gel application on the dental surfaces of the treated teeth (UR5-UL5; LR5-LL5). (**a**) The initial application of BlancOne ULTRA^+^ gel was on the outer surface of the treated tooth by pushing the whitening gel syringe piston in a thin layer (2–3 mm); (**b**) a clinical photo shows the complete application of a thin layer of the bleaching gel on the outer surfaces of the upper treated teeth and the process of completing the gel application on the lower-treated teeth.

**Figure 8:**
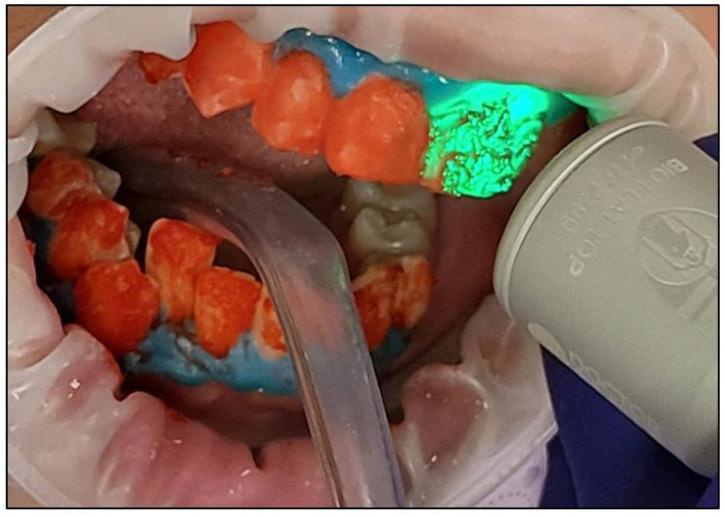
A clinical photo shows the photoactivation process of the BlancOne ULTRA^+^ gel with λ 450 nm photonic energy delivered with a flattop handpiece, indicating the laser light-gel interaction.

**Figure 9:**
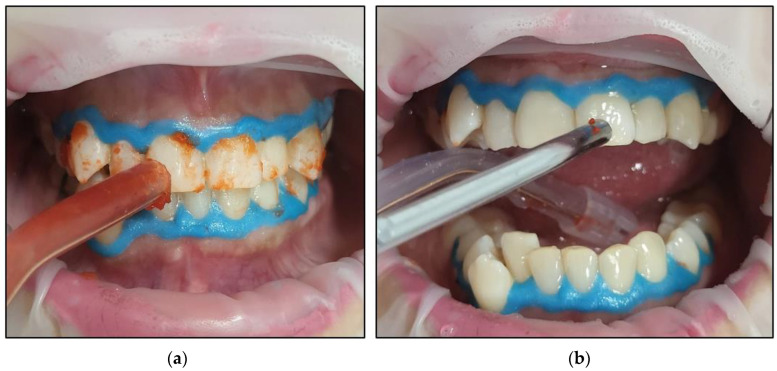
Clinical photos illustrate the technique that was utilised for removing the bleaching gel. (**a**) the first step was using dental suction; (**b**) the second step was washing the dental surfaces of the treated teeth and drying them with cotton rolls.

**Figure 10:**
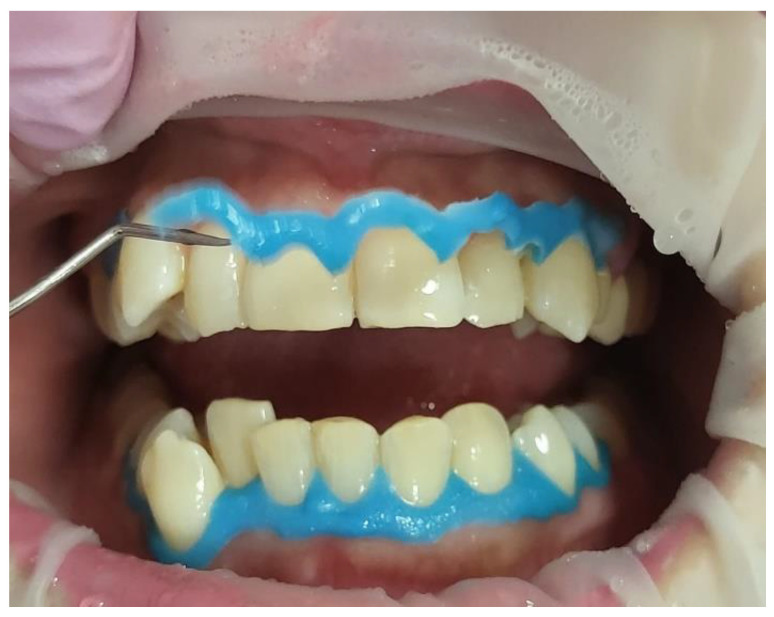
A clinical photo showing the technique used for removing the gingival barrier with a dental scaler after completing the three whitening cycles.

**Figure 11:**
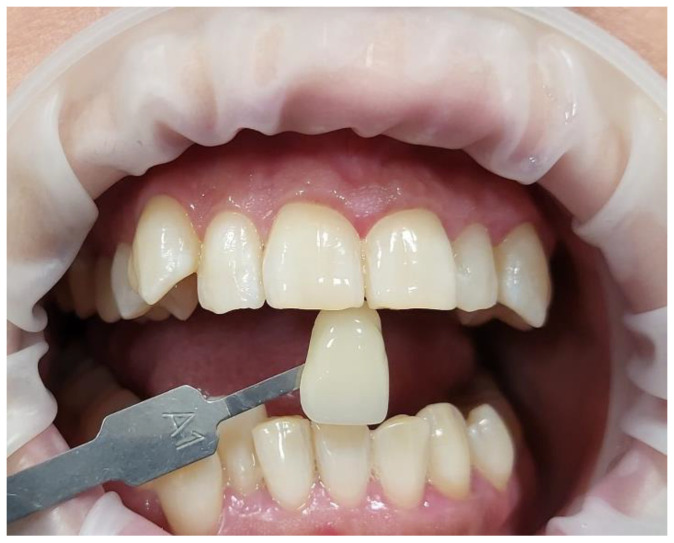
A clinical photo shows significant results in obtaining shade colour “A1” immediately after three whitening cycles.

1. All of the health and safety protocols were implemented. The patient, the operator, and the dental nurse wore the appropriate protective eyewear for the λ 450 nm laser during the entire bleaching treatment;
2. The BlancOne ULTRA^+^ whitening gel was applied on the outer surface of the following teeth that required whitening (UR5-UL5 and LR5-LL5) by gently pushing the gel syringe piston in a thin layer ~2–3 mm in thickness (Figure 7);
3. The buccal surfaces of the treated teeth were photoactivated with a λ 450 nm laser with photonic energy delivered with a flattop handpiece immediately after the gel was applied for 15 s (Figure 8). Figure 8 clearly shows the laser’s light interaction with the gel, indicating an effective photoactivation process whereby photonic energy is equally distributed on the buccal surface of the treated tooth via a flattop delivery handpiece.
4. Whitening Cycle Protocol
  - The bleaching gel was applied to the teeth in a thin layer;
  - Fifteen seconds irradiation time per spot was used to photoactivate the bleaching gel with λ 450 nm photonic energy delivered using a flattop handpiece. There were five spots per arch. The time required for the photoactivation of both arches was ~three minutes (min);
  - The gel was rested on the teeth’s surface for 8 min between each whitening cycle.
  - The gel was removed using dental suction (Figure 9a), and then the dental surfaces were thoroughly rinsed (Figure 9b) with continuous suction. Then, all the teeth were dried with cotton rolls. This process was repeated at the end of each cycle. Freshly prepared gel applications for each whitening cycle were performed;
  - A new layer of whitening gel was applied;
  - The whitening cycle was repeated three times in one session with an 8 min gel resting period (thermal relaxation) between each cycle.
5. After three whitening cycles, the gingival protective barrier was gently removed with a scaler, as shown in Figure 10;
6. Figure 11 shows a clinical photo of case #1 immediately after three whitening cycles, obtaining the A1 shade colour;
7. The dental hypersensitivity/pain was reported based on the patient’s self-reporting of the VAS at pre-treatment (T0), during the three whitening cycles, at the end of three whitening cycles (T1), 24 h (h) (T2) and 48 h (T3) post-treatment and 8-month follow-up (T4);
8. The colour shade at T0, T1, and T4 were recorded by two experienced independent assessors;
9. The gingival irritation was assessed during the whitening cycles, at T1 in the clinic, and at T2 and T3 via a telephone call;
10. The patient’s treatment satisfaction was evaluated at T1 at the clinic and at T2 and T3 via a telephone call;
11. Post-treatment instruction leaflets were provided to all the patients to ensure good treatment maintenance.

### 2.6. Photoactivation Protocol and Laser Dosimetry

The diode laser device that was utilised in the study (Wiser 3, Doctor Smile-Lambda, Vincenza, Italy) emits λ 450 nm photonic energy to activate the BlancOne ULTRA^+^ gel. The handpiece of the laser device is based on a flattop beam profile (Figure 9), delivering photonic energy to activate the applied whitening gel on the buccal surfaces of the treated teeth.

**Figure 12:**
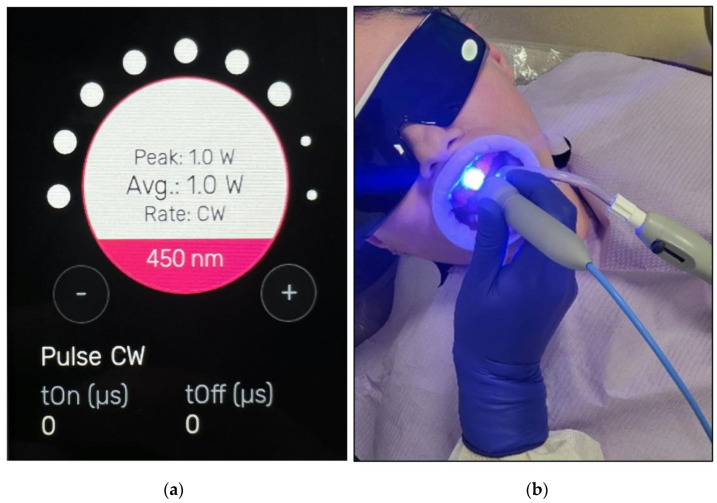
(**a**) The utilised laser dosimetry protocol for photoactivation appearing on the device panel; (**b**) the delivery of λ 450 nm photonic energy with the flattop handpiece during the photoactivation process of BlancOne ULTRA^+^.

Figure 12a shows the utilised photoactivation parameters displayed on the laser device, whereas Figure 12b shows a clinical photo illustrating the application of the flattop handpiece while delivering 450 nm photonic energy to photoactive the whitening gel.

Table 2 shows the details of the laser device specifications, the dosimetry of photoactivation, and the treatment protocol.

### 2.7. Outcome Assessment Tools

#### 2.7.1. Colour Shade Guide

The arrangement and classification of the VITA classical family shades A1-D4^®^ are as follows: A1–A4 (reddish-brownish); B1–B4 (reddish-yellowish); C1–C4 (greyish shades); and D2–D4 (reddish-grey). 

This standard shade guide was utilised in our study to determine and evaluate the colour shade of the buccal surfaces of the treated teeth at pre-treatment (T0), the dental colour change at the end of each whitening cycle (T1), and at the 8-month follow-up timepoint (T4). As the standard VITA shade guide is a subjective visual assessment method, two experienced independent assessors who were uninvolved in the study determined the final colour shade for all the timepoints to reduce the bias and discrepancies.

We removed any influencing factors, such as lipstick and cosmetic makeup. The operator held the shade sample tooth as close to the patient’s tooth as possible (Figure 11). 

The shade choice was quickly decided, and the first decision was accepted by the two experienced independent assessors since the eyes begin to tire after ~5–7 s.

Calibration of the VITA Easyshade^®^ machine was conducted each time before shade determination [45].

#### 2.7.2. Visual Analogue Scale

**Figure 13:**
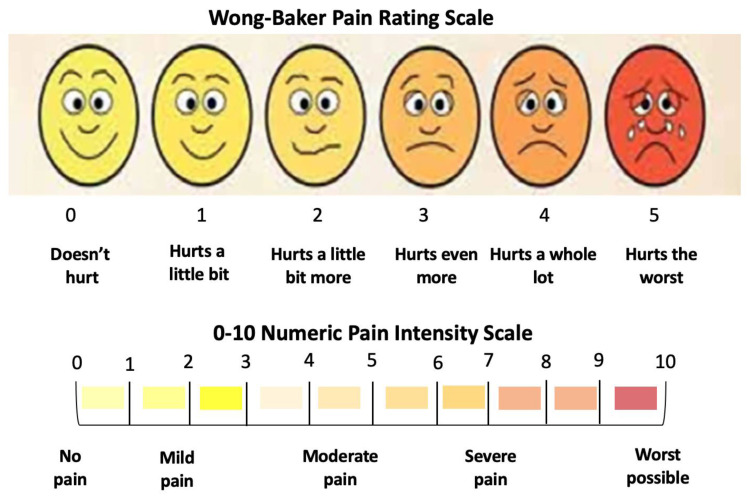
Wong–Baker Pain Rating Scale.

The patients’ self-reported data on pain and tooth hypersensitivity were evaluated using the visual analogue scale (VAS), which is a golden standard assessment tool for pain [46]. We utilised the Wong-Baker faces scoring scale and quantitative numeric pain intensity to record the variables’ scores, ranging from “0”—no pain to “10”—worst possible pain (Figure 13). The patient was familiarised with this assessment tool at the consultation appointment.

In order to evaluate the tooth hypersensitivity, an external stimulus was applied on the treated teeth using a triple syringe providing evaporative blowing for 3 s on the upper central incisors at a distance of 1 cm. This is a chairside technique was utilised to assess the tooth hypersensitivity of the bleach-treated teeth [47].

The scoring of pain and dental hypersensitivity were recorded as follows: T0, during and at T1 (at the clinic), T2 and T3 via a telephone call, and T4 at the clinic.

#### 2.7.3. Löe and Silness Gingival Index

The Löe and Silness gingival index (GI) scale [48] is based on the following gingival inflammation scoring: 0: normal gingiva; 1: mild inflammation: slight change in colour, slight oedema, no bleeding on probing; 2: moderate inflammation: redness, oedema, and glazing, or bleeding on probing; 3: severe inflammation: marked redness and oedema, tendency towards spontaneous bleeding. The variable was recorded during the whitening cycles, T1 at the clinic, and T2 and T3 via a telephone call.

#### 2.7.4. Patient Satisfaction

**Figure 14:**
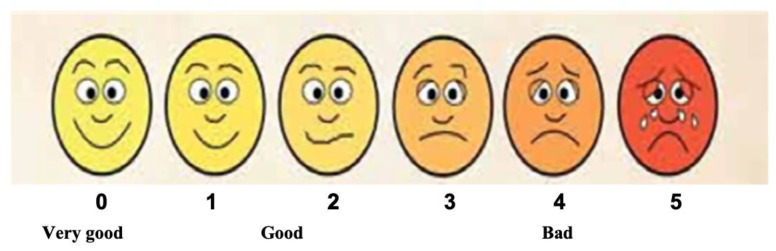
Modified Wong Baker faces scale used to evaluate patient treatment satisfaction, ranging from “0”, meaning very good, to “5”, meaning bad (adapted from Hanna et al., 2016, permission obtained [49]).

We utilised the modified Wong-Baker faces rating scale [49] to evaluate patient treatment satisfaction. It is based on the patient’s self-reporting scoring, ranging from “0”, which means “very good”, to “4–5”, which means bad (Figure 14). This variable was recorded at T1 (clinic) and T2 and T3 via a telephone call.

### 2.8. Statistical Analysis

Percentage is a statistical tool used to express the relative amounts of increase or decrease in a standardised ratio comparison. It is a descriptive analysis of relatively simple calculations that provide a basic picture of what the data looks like overall and shows proportions. The change in dental colour outcome values (quantitative variables) recorded at T0 and at different timepoints (T1, T2, T3, and T4) were expressed as percentages %. The statistical test for this calculation is called the z-test (one-sided) for the equality of two percentages using independent samples. A 5% level of significance is commonly used in statistics because it provides a balance between being too conservative and too liberal in accepting or rejecting a null hypothesis. 

The mean is a statistical tool that is calculated by adding the values in the dataset together and then dividing this by the number of added values.

## 3. Results

Our findings were significant in achieving optimal teeth whitening outcomes with no adverse effects. Additionally, our laser dosimetry and treatment protocols were valid and robust enough to ensure safety and patient satisfaction.

### 3.1. Demographic Characteristics

All of the six cases were females, with a mean age of 36.7-year-old. 50% of the subjects were smokers with a mean 13 cigarettes per day. None of the subjects had any systematic diseases (fit and healthy), and none of the subjects opted out. All of the subjects followed the treatment and follow-up protocols. Table 3 shows the subject demographic characteristics.

### 3.2. Assessment of Whitening Outcomes

Our findings show substantial improvements in the dental colour shades of the patients compared with their initial shades, ranging from 2- to 10-fold based on the VITA classical A1-D4^®^ shade guide (VITA Zahnfabrik, Bad Säckingen, Germany).

The assessment was performed by the operator and an independent healthcare professional. The following findings showed an improvement in the dental colour shades at T1, and these were maintained at T4. Two of six cases (33.3%), the colour shade was reduced by seven-fold, two out of six cases (33.3%), the colour shade was reduced by three-fold; one out of six cases (16.6%), the colour shade was reduced ten-fold, and the remaining case had their dental shade reduced by two-fold (Table 4).

**Figure 15:**
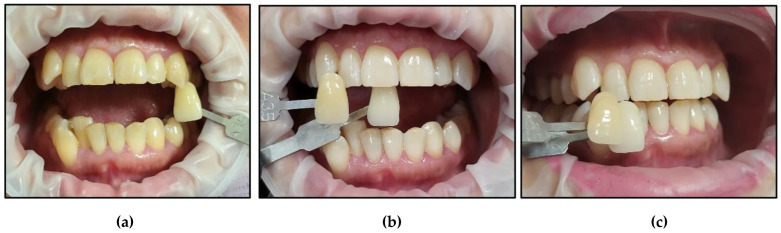
Clinical photos of case #1 illustrating the reduction in shade (10-fold) immediately after the bleaching treatment compared to the initial colour shade, and this was maintained at the 8-month follow-up. (**a**) Pre-treatment (A3.5); (**b**) immediately after bleaching treatment (A1); (**c**) at the 8-month follow-up (A1).

**Figure 16:**
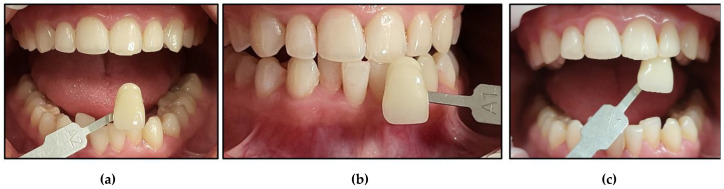
Clinical photos of case #2 illustrating reduction in shade (3-fold) immediately after the bleaching treatment compared to the initial colour shade, and this was maintained at the 8-month follow-up. (**a**) Pre-treatment (A2); (**b**) immediately after bleaching treatment (A1); (**c**) at the 8-month follow-up (A1).

**Figure 17:**
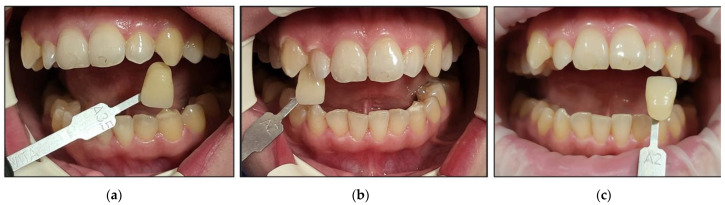
Clinical photos of case #3 illustrating the reduction in shade (7-fold) immediately after the bleaching treatment compared to the initial colour shade, and this was maintained at the 8-month follow-up. (**a**) Pre-treatment (A3.5); (**b**) immediately after bleaching treatment (A2); (**c**) at the 8-month follow-up (A2).

**Figure 18:**
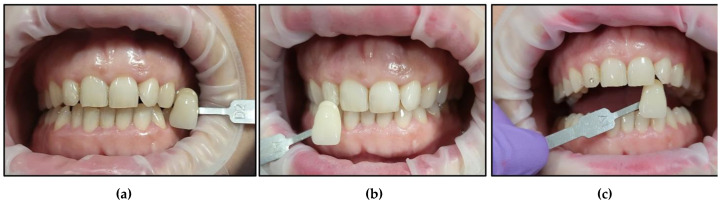
Clinical photos of case #4 illustrating the reduction in shade (2-fold) immediately after the bleaching treatment compared to the initial colour shade, and this was maintained at the 8-month follow-up. (**a**) Pre-treatment (D2); (**b**) immediately after bleaching treatment (A1); (**c**) at the 8-month follow-up (A1).

**Figure 19:**
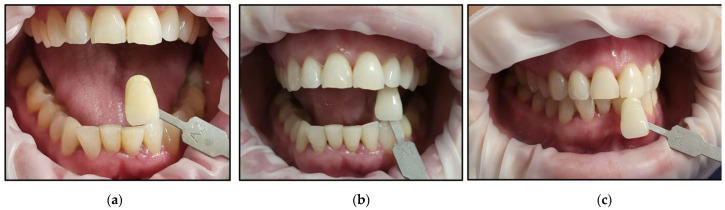
Clinical photos of case #5 illustrating the reduction in shade (3-fold) immediately after the bleaching treatment compared to the initial colour shade, and this was maintained at the 8-month follow-up. (**a**) Pre-treatment (A2); (**b**) immediately after bleaching treatment (A1); (**c**) at the 8-month follow-up (A1).

**Figure 20:**
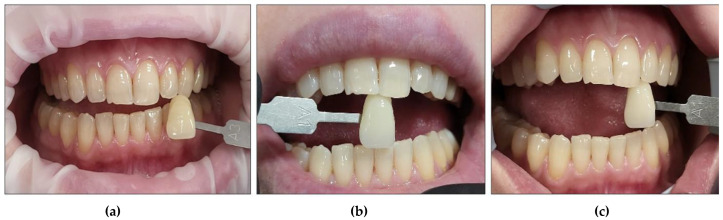
Clinical photos of case #6 illustrating the reduction in shade (7-fold) immediately after the bleaching treatment compared to the initial colour shade, and this was maintained at the 8-month follow-up. (**a**) Pre-treatment (A3); (**b**) immediately after bleaching treatment (A1); (**c**) at the 8-month follow-up (A1).

It is noteworthy that all the patients followed only their regular dental hygienic habits during the follow-up periods without using any at-home bleaching products. We outlined below the clinical outcomes of dental colour shades for all six patients at T1 and T4 compared to T0 (Figure 15, Figure 16, Figure 17, Figure 18, Figure 19 and Figure 20):

Case #1

Case #2

Case #3

Case #4

Case #5

Case #6

### 3.3. Pain and Bleaching-Induced Tooth Sensitivity Assessment

We evaluated pain and tooth hypersensitivity using the VAS based on patients’ self-reported pain scoring during the bleaching treatment at T1, T2, T3, and T4. All the patients scored “0” on the VAS for pain or tooth hypersensitivity during the whitening cycles and at T1, using the external stimulus of an evaporative blowing triple syringe for 3 s on the upper central incisors at a distance of 1 cm. Additionally, the patients reported no pain or dental hypersensitivity (they scored “0” on the VAS) at T2 and T3 postoperatively, and none of them required analgesics. At T4, no patients reported dental hypersensitivity.

### 3.4. Assessment of Gingival Irritation

None of the patients developed any gingival inflammation. All the patients scored “0” based on the GI during the whitening cycles, T1, T2, and T3. This was well demonstrated in the clinical photos of the six cases (Figure 15, Figure 16, Figure 17, Figure 18, Figure 19 and Figure 20). The utilisation of a gingival barrier to protect the gingivae of the treated teeth and the operator’s expertise played a vital role in minimising gingival inflammation. 

### 3.5. Evaluation of Patient Satisfaction

All the patients were satisfied with the outcome of the bleaching treatment. An independent healthcare professional assessed the patients’ satisfaction using the “modified Wong-Baker faces pain rating scale”, whereby 90% scored “0”, and 10% scored “1”. Significant patient satisfaction for the bleaching treatment was reported in all of the six patients.

## 4. Discussion

The present case series study evaluated the tooth whitening outcomes utilising, for the first time, a λ 450 nm blue laser delivered with a flattop beam profile handpiece to photoactivate the unique composition bleaching agent, “BlancOne ULTRA^+^”, which achieved optimal clinical aesthetic outcomes with sustainability of the dental colour shade at T4, ensuring safety and maximising patient treatment satisfaction. Hence, the H_0_ was rejected.

Our interpretation of the results and perspective on previous studies are outlined below.

### 4.1. Appraising the Optimised Whitening Outcomes

Our findings showed a significant colour shade improvement in all the treated teeth for all six cases immediately after the in-office bleaching treatment, and this was sustained at the 8-month follow-up timepoint. This is indicative that our choice of the bleaching agent (BlancOne ULTRA^+^), photoactivation time, laser light source, application time of the whitening agent, laser photoactivation dosimetry with the flattop delivery system, and treatment protocols were collectively effective and safe in achieving a sustainable colour change outcome with no adverse effect. This could explain why the current scientific literature showed that in-office bleaching treatment improves the colour of the enamel but is not sustained after six months [50,51]. 

#### 4.1.1. Evaluating the Concept of H_2_O_2_ Concentration in the Bleaching Agent 

H_2_O_2_ concentration between 25% and 40% plays a vital role to maximise the bleaching process and achieve optimal change in the tooth colour shade [52]. The oxidising ability of H_2_O_2_ may be responsible for the reduction in dentinal organic components when the bleaching product is applied directly to the dentine [53] 

Bleaching agent penetration mainly occurs because of low molecular mass and the ability to denature proteins, increasing ion movement through the tooth’s structure [54], but it is also influenced by tooth density and dentinal tubule diameter [55].

A study conducted by Polydorou et al., 2018 [56] showed the highest increase in enamel microhardness after bleaching with 40% H_2_O_2_. On the other hand, a study conducted by de Souza Costa et al., 2010 [57] highlighted the correlation between H_2_O_2_ concentration and its application time. They concluded that H_2_O_2_ at a concentration of 38% for an application time of 45 min led to irreversible pulp damage in the lower incisors but not in the premolars, indicating that the size of the tooth is another factor to consider to ensure safety and avoid tooth hypersensitivity [39]. 

Its formulation contains three natural photoactivators in synergy with the λ 430–490 nm wavelength range, which could cause the more efficient release of free radicals and singlet oxygens to allow faster and more effective bleaching results. 

#### 4.1.2. Laser vs. LED Assessment in Dental Bleaching

Some authors have questioned the use of lasers for the activation of tooth whitening gels due to increased hypersensitivity and a non-significant difference when a bleaching agent is not laser-activated [58]. This might be attributable to the laser used for the activation of the bleaching gel. On the other hand, a clinical investigation indicated that the use of a diode laser resulted in less dental and gingival sensitivity compared with non-activated bleaching agents [59]. 

It was shown that laser-assisted bleaching is more effective than an LED-activated system in terms of changes in chroma and luminosity [60] and hence the results of our study were optimal in colour shade change because we utilised λ 450 nm blue laser. Also, the delivery system that we employed in our study was based on flattop beam profile and hence the laser photonic energy was equally distributed over a 1 cm^2^ surface area minimising light scattering. In contrast, an LED light source can cover a large area at a shallow penetration depth [42]. Our study parameters used wavelength-specific high-absorbing pigments.

#### 4.1.3. Bleaching Application Time

BlancOne ULTRA^+^ requires a short photoactivation time (15 s) and 8 min resting application time on the tooth surface between each cycle with a low number of treatments (three whitening cycles). It works through a process of chemical photoactivation of the gel using specific wavelengths of light, which in turn can accelerate the release of free radicals and singlet oxygens [61]. This whitening agent content contributed to achieving optimal outcomes with no adverse effects. 

The link between bleaching application time and enamel morphological changes is well-documented in the literature [56,62,63]. A study conducted by Polydorou et al., 2018 [56] emphasised that the long-term use of tooth bleaching agents and the amount of H_2_O_2_ used do not seem to be the most important factors concerning the alterations of enamel surface properties. Instead of this, the application time of the bleaching agent used for each method seems to play an important role. This is different to our study, whereby our study employed a short application time. Hence, we anticipated no morphological changes in the dental hard tissues. This was supported by a study conducted by Bistey et al., 2007 [62], which concluded that the effects on the enamel structure due to bleaching were time-dependent, suggesting that an application time higher than 60 min could cause considerable effects. A study conducted by Mondelli et al., 2009 [63] agreed that application time is an important factor concerning the effect of bleaching agents on the enamel.

Additionally, the literature revealed a link between bleaching application time and its impact on dental pulp toxicity. An in vitro study conducted by Soares et al., 2016 [64] indicated that the standard in-office bleaching contained 35% H_2_O_2_ gel applied for 45 min, resulting in a rapid and effective whitening outcome, but could produce strong oxidative stress on the pulp cells associated with an intense reduction in cell viability. However, if the bleaching application time was reduced, there was less toxicity to the pulp viability with satisfactory improvements in the colour shade due to a reduction in the trans-enamel and trans-dentinal cytotoxicity to the pulp cells. This is a strong indication that a very short bleaching application time in the present study had no harmful impact on pulp cell viability.

#### 4.1.4. Temperature

Temperature accelerates the reaction rate of photoactivation, leading to a quicker whitening session. For every 10 °C temperature increase, the rate of the chemical reaction is doubled. An increase in temperature could also cause dental sensitivity and reversible pulpitis in patients if it is too high. i.e., >53 °C. However, if the photoactivation time is short [65] and the increase in the intrapulpal temperature remains under the safety threshold of 5.5 °C, this would indicate a very safe bleaching strategy [66,67].

An in vitro study conducted by Morsi et al., 2020 [68] evaluated intrapulpal temperature during λ 445-nm diode laser irradiation with different parameters. The study findings showed that a power output between 0.2 W and 1 W in the continuous emission mode (CW) with an up to 60 s irradiation time; 1.5 W, CW up to 15 s; 2 W, CW, 10 s; and 2.5 W, CW, 10 s are biologically safe parameters for the dental pulp during dentine hypersensitivity treatment [67]. 

Our findings are in agreement with the above-mentioned studies, as our photoactivation parameters were as follows: λ 450 nm; 1 W power output; CW; 15 s. Even our irradiation exposure time was much less than 60 s, indicating that our study parameters were safe without jeopardising pulp vitality. 

The findings of the present case series are in agreement with the findings of a study conducted by Al-Hamd et al., 2023 [69], which confirmed that the utilisation of short wavelength diode lasers, such as λ 405 nm or λ 450 nm, in vital teeth bleaching revealed a minimal temperature rise of 1.1 °C, the best colour shade results, improved microhardness and, moreover, did not affect external tooth roughness. 

### 4.2. Evaluation of Post-Operative Complications

Dental hypersensitivity is the main undesirable effect caused by dental bleaching, inducing pain and discomfort during and after the procedure [70] due to the pulpal sensory afferents expressing transient receptor potential cation channel subfamily A number 1 (TRPA1) and direct activation of intradental nerve activity via TRPA1 contributes to the mechanism of bleaching sensitivity pain. These nervous fibres are myelinated and responsible for the fast transmission of acute pain through A-delta nervous fibres [71]. 

Our results revealed that patients’ self-reported scores were “0” for pain and dental hypersensitivity during the whitening cycles T1, T2, T3, and T4. 

In lieu of the above findings, patients self-reported no pain or hypersensitivity at T1 when the treated teeth were subjected to the stimulus of an evaporative blowing triple syringe for 3 s on the upper central incisors from a distance of 1 cm. This underscores that our treatment protocol collectively contributed to pain alleviation via the following: bleaching agent composition, a short photoactivation time (15 s); a short bleaching resting time (8 min between whitening cycles); the flattop beam profile delivery system; the λ 450 nm laser light; and the experienced operators.

On reflection, the λ 450 nm blue laser provoked antihyperalgesic and antiallodynic effects by blocking the delta A and C fibres, causing the hyperpolarisation of the nervous fibre, preventing transduction, blocking nervous transmission, and promoting pain alleviation [72]. As lasers have unique properties of monochromaticity and coherence, they succeed in being more potent anti-inflammatory and analgesic biomodulators [73,74,75,76] compared to LEDs.

Neutral formulations of the bleaching agent have been demonstrated to be less destructive to enamel because of the alkaline salt that adheres to the enamel surface, limiting direct contact between hydrogen peroxide and the enamel. Alkaline and neutral bleaching agents have been shown to not only be less detrimental to the enamel but are also more effective whitening agents [77]. Moreover, the whitening agent acts as a desensitising vehicle to reduce the symptoms of dental hypersensitivity by occluding the patient’s dentinal tubules during the whitening cycles [78]. Hence, our study evaluated the pain and tooth sensitivity at T1. Additionally, the latter symptoms were evaluated at T2 and T3 (24 h and 48 h post-bleaching treatment, respectively), as well as at the 8-month follow-up, ensuring the sustainability of the optimal outcome. 

In lieu of the above-mentioned notes, our study answered how we could overcome the shortfalls and the scientific debate on dental hypersensitivity in vital teeth whitening associated with a high concentration of H_2_O_2_ and a long exposure time to dental structures, and this sensitivity during bleaching is typically moderate [79,80].

None of the patients developed any gingival inflammation. All of the patients scored “0” based on the GI, which is a robust assessment tool [81]. All of this is demonstrated well in all patient’s clinical photos. The utilisation of a gingival barrier to protect the gingivae and the operator’s experience also played an important role in minimising gingival inflammation. 

### 4.3. Bleaching Treatment Safety, Feasibility, and Satisfaction

All the patients were satisfied with the outcome of the bleaching treatment. An independent healthcare professional assessed the patients’ satisfaction using the “modified Wong-Baker faces pain rating scale”, where 90% scored “0” and 10% scored “1”. Significant satisfaction with the bleaching treatment was reported in all of the six patients. This is in agreement with the scientific literature that asserts that vital in-office tooth bleaching with optimal whitening outcomes and without post-bleaching tooth sensitivity can have a significant impact on patient’s QoL [3,82]. 

### 4.4. Study Limitations and Future Directions

The limitations of the study are as follows: (1) an uncontrolled longitudinal observational study on a series of subjects receiving the same intervention (i.e., there was no control/sham group; (2) a low number of treated patients; (3) represents level IV evidence-based medicine; (4) a little statistical validity due to a lack of the control group to compare the outcomes; (5) a lack of quantitative assessment tool.

Despite the above-mentioned study limitations, significant results demonstrated the efficacy of λ 450 nm laser photoactivation of a bleaching agent with a unique chemical composition in a short amount of time (15 s), leading to optimisation of the whitening outcomes and ensuring safety in terms of tooth sensitivity, as well as answering the controversy and discrepancy in the current scientific literature. This offers a useful guide for dental clinicians who perform vital in-office tooth whitening and paves a future perspective for extensive research with large datasets. 

## 5. Conclusions

Our results, for the first time, reported optimal dental colour shade improvements and sustainability at T4 without post-bleaching adverse effects (dental hypersensitivity), ensuring dental pulp safety and good patient treatment satisfaction. Furthermore, our findings indicate that the utilisation of a λ 450 nm blue laser delivered with a flattop beam profile could exhibit photobiomodulatory effects in reducing inflammation and blocking the transduction of the stimulus, leading to pain alleviation. This profoundly eliminated dental sensitivity during the treatment cycles at T1, T2, T3, and T4. This provides a useful guide for investigators to conduct extensive studies with large datasets.

## Figures and Tables

**Table 1:** Brief description of the utilised bleaching agent, its mode of use, and the treatment protocol [40].

Product Name	% Hydrogen Peroxide	pH	Working Type Mode	Photoactivation Wavelength	Photoactivation Time	Gel Resting Time between Each Cycle	Number of Applications/ Sessions
BlancOne ULTRA^+^	35	5.5	light	430–490 nm	15 s	8 min	3

**Table 2:** Laser device specifications, photoactivation parameters, and treatment protocol.

Device Specifications	Manufacturer	Doctor Smile-Lambda-Italy
	Model identifier	Wiser 3
	Emitter type	Diode laser
	Medical/laser class	IV
	Beam delivery system	Fibre
	Probe design	Single probe
	Beam profile	Flattop
	Beam divergence full angle	0°
Irradiation Parameters	Wavelength (nm)	450
	Therapeutic power output (W)	1
	Emission mode	CW
	Beam spot size at target (cm^2^)	1
	Irradiance at target (W/cm^2^)	1
	Energy per spot (J)	15
	Total energy	150
	Fluence (J/cm^2^) per point	15
	Irradiation time (s)	15
Treatment Protocol	Total number of irradiated points	10
	Laser-tissue distance	~2 cm (No loss of energy due to the unique properties of the flattop delivery system)
	Application technique	Static
	Total number of treatments/sessions	3
	Frequency of session	Once

**Table 3:** Demographic characteristics of the subjects.

Case #	Gender	Age	Systemic Disease	Medication	Smoking Status	No. of Cigarettes/Day
1	F	43	Nil	Nil	Yes	20
2	F	25	Nil	Nil	Yes	15
3	F	37	Nil	Nil	No	-
4	F	43	Nil	Nil	Yes	5
5	F	38	Nil	Nil	No	-
6	F	34	Nil	Nil	No	-

**Table 4:** Dental shade colour improvement immediately after the bleaching treatment (T1) and at the 8-month post-treatment timepoint (T8) compared to the initial shade colour prior to bleaching treatment (T0). This table also shows the colour shade fold improvement. All the shades were assessed using the Vita Easyshade^®^ digital spectrometer.

Case #	Colour Shade at T0	Colour Shade at T1	Colour Shade at T4	Colour Shade Fold Improvement at T1 and Maintained at T4 compared to T0
1	A3.5	A1	A1	10
2	A1	A1	A1	3
3	A3.5	A2	A2	7
4	D2	A1	A1	2
5	A2	A1	A1	3
6	A3	A1	A1	7

## Data Availability

All the data are included in the text.
